# Extracellular Vesicles as Tools for Crossing the Blood–Brain Barrier to Treat Lysosomal Storage Diseases

**DOI:** 10.3390/life15010070

**Published:** 2025-01-09

**Authors:** Giovanni Lerussi, Verónica Villagrasa-Araya, Marc Moltó-Abad, Mireia del Toro, Guillem Pintos-Morell, Joaquin Seras-Franzoso, Ibane Abasolo

**Affiliations:** 1 Clinical Biochemistry, Drug Delivery & Therapy (CB-DDT), Vall d’Hebron Institute of Research (VHIR), 08035 Barcelona, Spain; giovanni.lerussi@vhir.org (G.L.); veronica.villagrasa@vhir.org (V.V.-A.); marc.molto@vhir.org (M.M.-A.); guillem.pintos@vhir.org (G.P.-M.); joaquin.seras@vhir.org (J.S.-F.); 2 Networking Research Center on Bioengineering, Biomaterials and Nanomedicine (CIBER-BBN), 08035 Barcelona, Spain; 3 Institute of Advanced Chemistry of Catalonia (IQAC), Centro Superior de Investigaciones Científicas (CSIC), 08034 Barcelona, Spain; 4 Pediatric Neurology Unit, Hospital Universitari Vall d’Hebron and MetabERN, 08035 Barcelona, Spain; mireia.deltoro@vallhebron.cat; 5 Networking Research Center on Rare Diseases (CIBERER), 08035 Barcelona, Spain

**Keywords:** blood–brain barrier, drug delivery, enzyme replacement therapy, exosomes, extracellular vesicles, lysosomal storage diseases, neurodegenerative diseases, protein delivery

## Abstract

Extracellular vesicles (EVs) are nanosized, membrane-bound structures that have emerged as promising tools for drug delivery, especially in the treatment of lysosomal storage disorders (LSDs) with central nervous system (CNS) involvement. This review highlights the unique properties of EVs, such as their biocompatibility, capacity to cross the blood–brain barrier (BBB), and potential for therapeutic cargo loading, including that of enzymes and genetic material. Current therapies for LSDs, like enzyme replacement therapy (ERT), often fail to address neurological symptoms due to their inability to cross the BBB. EVs offer a viable alternative, allowing for targeted delivery to the CNS and improving therapeutic outcomes. We discuss recent advancements in the engineering and modification of EVs to enhance targeting, circulation time and cargo stability, and provide a detailed overview of their application in LSDs, such as Gaucher and Fabry diseases, and Sanfilippo syndrome. Despite their potential, challenges remain in scaling production, ensuring isolation purity, and meeting regulatory requirements. Future developments will focus on overcoming these barriers, paving the way for the clinical translation of EV-based therapies in LSDs and other CNS disorders.

## 1. Introduction

Extracellular vesicles (EVs) are nanosized, membrane-bound vesicles that are released by all cell types into the extracellular space. EVs are categorized based on their biogenesis, biological function, and size, with the primary types being exosomes (30–150 nm in diameter), microvesicles (MVs) (100–1000 nm), and apoptotic bodies (1–5 μm) [1]. They carry a diverse array of biomolecules, including lipids, proteins, and nucleic acids, and serve as vehicles for intercellular communication. By influencing recipient cells, EVs modulate a wide range of biological processes in both neighboring and distant tissues [2].

The cellular origin and mechanisms of EV formation contribute to the significant heterogeneity in EV cargo. This diversity positions EVs as valuable biomarkers for physiological and pathological states, as their contents often reflect the condition of their cell of origin [3]. According to the MISEV2018 [4], EVs can also be classified into small EVs (sEVs), typically ranging from 30 to 200 nm in diameter and, thus, containing mainly exosomes, and large extracellular vesicles (lEVs), which are generally 150 to 1000 nm in size. sEVs are involved in cell-to-cell communication and the transfer of proteins, lipids, and RNA molecules, whereas lEVs play roles in similar processes but are often associated with larger cargo, including cellular debris and organelle fragments, and are involved in immune responses and tissue repair [5]. Proteins like SNAREs, ESCRT, tetraspanins (CD9, CD63, CD81), ADAMs and Rab are valid biomarkers for sEVs, while lEVs display an increased expression of cytokinesis, ribosomal, mitochondrial and nuclear proteins [6].

Due to their capacity to transport bioactive molecules, EVs are being increasingly studied as carriers for therapeutic agents, including proteins [7]. A particularly promising aspect of EVs is their inherent ability to traverse biological barriers, notably the blood–brain barrier (BBB), which poses a significant obstacle in treating central nervous system (CNS) disorders [8]. Research has shown that EVs can be engineered through genetic modifications of secreting cells to optimize therapeutic delivery, including targeted delivery to difficult-to-reach tissues such as CNS [9]. Furthermore, post-isolation loading techniques, including chemical or mechanical methods, such as sonication, saponification, or simple incubation, enable the incorporation of a variety of therapeutic molecules, including siRNA, enzymes, and chemotherapeutics [7]. These unique properties of EVs are leading to their emergence as promising vectors for the treatment of CNS-related diseases.

This review aims to explore the therapeutic potential of EVs, specifically, in lysosomal storage disorders (LSDs), a group of rare diseases with substantial CNS involvement.

## 2. Challenges in the Treatment of Lysosomal Storage Diseases (LSDs)

LSDs are a group of rare genetic disorders, caused by mutations that disrupt the normal production or function of proteins essential for lysosomes [10]. While treatments are available for a limited number of LSDs, most therapeutic options remain ineffective in addressing CNS damage [11]. This underscores the urgent need for alternative therapeutic pathways to improve outcomes for LSD patients [10].

### 2.1. Etiology, Clinical Manifestations and Treatment of LSD

LSDs encompass more than 70 different pathologies, most of them caused by single gene mutations. The majority of these disorders follow an autosomal recessive inheritance pattern; however, a few, such as Fabry disease (FD), Hunter syndrome and Danon disease, are inherited in an X-linked manner [12]. Mutations lead to deficiencies or the dysfunction of proteins that are crucial for the multiple biological functions of the lysosome. These include enzymes, membrane proteins, transporters, and signaling or trafficking proteins that help lysosomal hydrolases interact with substrates and other organelles [10]. The accumulation of undegraded substrates within lysosomes initiates a pathogenic cascade, causing a range of clinical symptoms that affect multiple organs and systems [10,13].

Although LSDs are individually classified as rare diseases, their collective prevalence is notable, with a cumulative incidence estimated in approximately 1 in 5000/7500 births [14]. The advances in newborn screening programs worldwide [15], along with improved differential diagnostic methods for symptomatic patients [16], are continually reshaping our understanding of the prevalence and the incidence of these disorders. Table 1 presents a summary of the most prevalent LSDs, their associated gene mutations, accumulated substrates, and estimated incidence rates.

**Figure 1:**
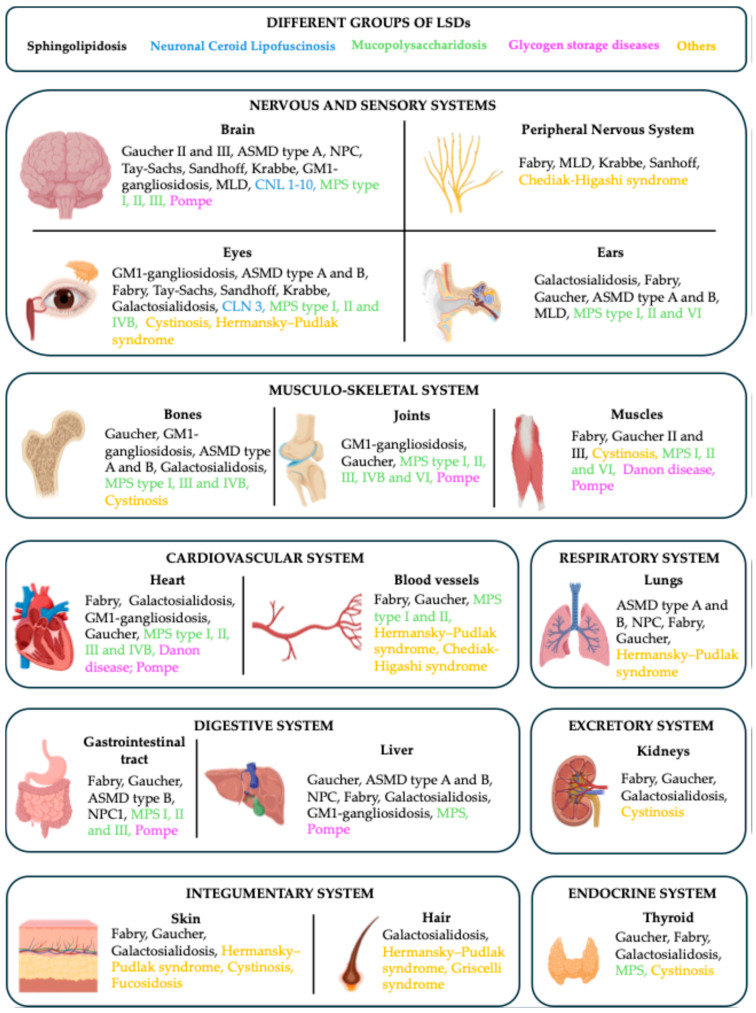
Multisystemic manifestations of lysosomal storage disorders (LSDs). Main disorders showing manifestations in various organs and tissues are indicated. The image does not aim to list all the manifestations of all the diseases, but instead, give an idea of the multisystemic nature of the LSDs.

Concerning the clinical manifestations in LSDs, not all cell types and systems in the body are equally affected. Factors such as the unique biochemistry of different cell types, redundancy in catabolic enzymes, varying substrate turnover rates and adaptive changes [10] lead to considerable variations in disease onset and severity (see Figure 1). The accumulation of substrates in cells and tissues gives rise to a range of clinical manifestations, affecting the neurological, visceral, ocular, hematological and skeletal systems, with some overlap in symptoms across different disorders. Even within a single disease, individuals may present with different symptom profiles, ages of onset and disease progression rates. Starting symptoms can range from early infancy to late adulthood, making some lysosomal disorders challenging to diagnose until the later stages of life. As an example, in FD, the first and most frequent clinical manifestations in pediatric patients were found to be neurological and gastrointestinal [17]; as the patients become adults and disease progresses, patients may develop kidney, cardiac and cerebrovascular complications [18].

**Table 1:** Most frequent lysosomal storage disorders (LSDs), with their mutated gene, accumulated substrates and their estimated incidence worldwide [12,19,20,21]. Diseases are grouped according to the type of compound accumulated.

Disease and Group of Diseases	Mutated Gene	Principal Accumulated Substrate	Estimated Incidence
Sphingolipidoses			
Gaucher disease (GD)	GBA	Glucosylceramide (or glucocerebroside)	1:30,000
Fabry disease (FD)	GLA	Globotriaosylceramide (Gb3)	1:40,000/120,000
Metachromatic leukodystrophy (MLD)	ARSA	Sulfatides	1:40,000/160,000
Krabbe disease (KD)	GALC	Galactosylceramide and psychosine	1:100,000
Niemann–Pick type C (NPC)	NPC1/NPC2	Cholesterol	1:150,000
Mucopolysaccharidoses (MPS)			
MPS type I	IDUA	Heparan sulfate, dermatan sulfate	1:100,000
MPS type IIIA	SGSH	Heparan sulfate	1:100,000
Glycogen storage diseases			
Pompe disease	GAA	Glycogen	1:40,000

**Figure 2:**
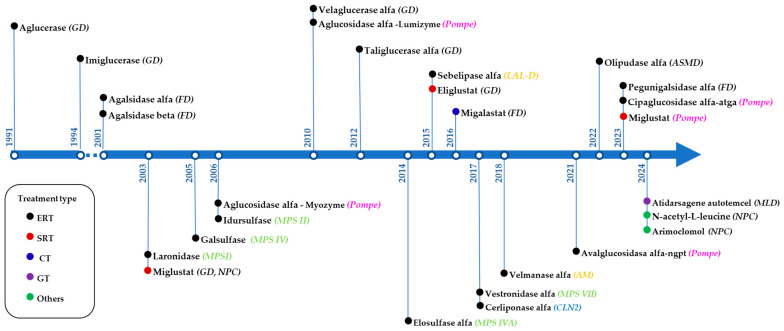
Timeline for the approval of LSD-specific therapies. For the sake of clarity, stem cell transplantation has not been included. Therapies: ERT—enzyme replacement therapy; SRT—substrate reduction therapy; CT—chaperone therapy; GT—gene therapy. Diseases: GD—Gaucher disease; FD—Fabry disease; MPS—mucopolysaccharidosis; NPC—Niemann–Pick type C; LAL-D—lysosomal acid lipase deficiency; ASMD—acid sphingomyelinase deficiency; CLN2; AM—alpha mannosidosis; MLD—metachromatic leukodystrophy. For the sake of clarity, stem cell transplantation has not been included in the scheme.

For the majority of LSDs, effective treatments are not available and treatments are mainly symptomatic and supportive. Among the specific therapies, the first approved drug for LSDs was Ceredase^®^ (Genzyme), a placentally derived β-glucocerebrosidase enzyme, to treat type 1 GD, in 1991. Since then, enzyme replacement therapies (ERTs), which involve the exogenous intravenous (i.v.) administration of recombinant proteins to restore the correct function of lysosomal enzymes, have become the most effective treatment strategy for LSDs, with twelve other ERTs approved during the last decades [22] (See Figure 2).

Other approved therapies, apart from ERT, include the following: hematopoietic stem cell transplantation (HSCT) [23], in vivo or ex vivo gene therapies, small drug molecules for substrate reduction therapies (SRT) [24], chaperone therapy (CT) [14], or the regulation of autophagy and proteostasis [10]. HSCT was the main therapeutic option for some LSDs before ERT became available, and it is still a useful strategy in certain diseases with CNS involvement, such as mucopolysaccharidosis (MPS) type I, Krabbe disease (KD) or metachromatic leukodystrophy (MLD), when gene therapy is not available, despite its association with high morbidity and mortality rates.

Among small pharmacological treatments, chaperone therapy with Migalastat and SRT with Eliglustat were approved for GD in 2002 and 2014, respectively. In 2015, the former was also approved for FD. These oral drugs are notable for their ability to cross the BBB, and some, like Miglustat, are potentially effective in the treatment of different LSDs. Up till now, gene therapy has received approval for only one LSD, metachromatic leukodystrophy (MLD), on March 2024, and its development is advancing more slowly than originally anticipated [25].

### 2.2. LSDs with Primary CNS Affection

At least 75% of LSDs present with progressive CNS manifestations in various degrees [26]. This neurological degeneration is mostly observed in infancy or childhood [27], leading to a significant decline in quality of life and premature death [28]. As mentioned before, no effective therapies are available for CNS; therefore, developing therapeutic strategies to delay or prevent neurological decline in LSD patients is urgent.

Neurological symptoms observed in patients depend on the age of onset but can include developmental delay or the regression of previously acquired abilities, abnormal eye movements, dementia, psychiatric issues, dysphagia, cerebellar ataxia, deafness, peripheral neuropathy, epilepsy, hypotonia, spasticity, myoclonus, abnormal head size, and stridor [22]. The sequence of events leading to neurodegeneration is complex and not only dependent on the accumulation of the primary storage material [19]. Initially, there is an accumulation of the non-degraded lysosomal substrate of the mutated protein or enzyme, followed by a secondary storage of other substrates. Depending on the specific diseases, this accumulation might affect different cells in the CNS. For instance, in GM1 gangliosidosis and KD, the first cells that are affected are neurons and oligodendrocytes, respectively, while in MPS III, astrocytosis and microgliosis are the initial triggers of disease progression [22,29]. Overall, alterations at the cellular level in the autophagy, endocytosis and exocytosis processes and mitochondrial dysfunction seem to be a common feature of the neurodegeneration in LSDs. These disruptions are associated with neuronal dysfunction, affecting axonal transport, synaptic vesicle processing, and other critical processes. In most LSDs, neurodegeneration is more common than a complete loss of neurons, with cell death typically occurring only in the later stages of the diseases. Exceptions include the selective loss of Purkinje neurons seen in Niemann–Pick type C (NPC) and the extensive cell loss observed in neuronal ceroid lipofuscinoses (NCL) [30,31].

Neuroinflammation is emerging as another trademark of LSDs. Substrate accumulation is associated with an activation or a perpetuation of an inflammatory state, contributing to neuronal death and degeneration [29,32,33]. The neuroinflammatory process in LSDs starts with a permeabilization of the lysosome membrane, leading to a leak of their cargo [34,35], having, as a consequence, a secondary accumulation of lysosomal proteins (cathepsin D and β-hexosaminidase among others) and storage materials inside and outside the cells [36]. The released materials act as damage-associated molecular patterns (DAMPs) which are sensed by others cells and trigger an immune response from glial cells and astrocytes, with the production of several types of different pro-inflammatory cytokines [32,33]. Specifically, elevated levels of TNFα and decreased levels of the anti-inflammatory cytokine IL10 were observed in *Npc1*^−/−^ mice brains [37,38]. Additionally, the upregulation of pro-inflammatory cytokines, such as IL1-α, IL1-β, IL6 and TNFα, was observed in *gba* null mice models of GD [39,40]. In the brain of MPS IIIA and IIIB mice, chemokines, such as Ccl3, and cytokines, such as IL1-β, were overexpressed [41,42]. Furthermore, CLN3Dex7/8 (NCL type 3) mouse microglia presented elevated levels of TNF-a, IL-1a, IL-9, and IL-15, among others [43].

LSDs have also been associated with other neurodegenerative diseases. This connection is not surprising, given the critical role of the autophagy lysosomal pathway (ALP) in degrading lipids, damaged organelles and eliminating protein aggregates, a hallmark of most neurodegenerative diseases [44]. A well-documented example is the relationship between Gaucher Disease (GD) and Parkinson’s diseases: carriers or individuals with GD type I have a higher risk of developing Parkinson’s disease. Additionally, many patients with this disease exhibit reduced β-glucocerebrosidase activity, along with an accumulation of glucosylceramide and related lipids, which may contribute to the stabilization of the neurotoxic α-synuclein [12].

As shown in Table 2, many of these disorders currently do not have approved treatments, or the approved ones are not effective in targeting CNS involvement. Developing drugs capable of crossing the BBB is essential, yet this remains a formidable challenge as nearly 100% of large therapeutic molecules and 98% of small therapeutic molecules are unable to penetrate this barrier [45].

In recent years, significant progress has been made in the development of drug delivery systems capable of crossing the BBB for the treatment of LSDs [11,47]. Despite these advancements, none have received regulatory approval yet. Among these innovative approaches, EVs have emerged as one of the most promising solutions. They show unique capabilities and potential, as will be discussed in the following sections.

## 3. Extracellular Vesicles for Delivery

Lipid-based nanoparticles, including liposomes and solid lipid nanoparticles, dominate the landscape of clinically approved organic nanoparticles [48]. These lipidic systems have been widely used in intravenous (i.v.) applications and they also protect delicate cargoes, such as mRNA, throughout various stages, from manufacturing to storage and delivery in vivo. Regarding biodistribution, EVs and negatively charged liposomes have shown a similar pattern upon i.v. administration [49,50]. EVs are rapidly cleared from circulation primarily due to the action of phagocytes, displaying an initial half-life of 20 min and a secondary half-life of 180 min [51]. Within 30 min post-administration, the majority of EVs localize to the liver, with smaller fractions distributed to the spleen, kidneys, and lungs. The tissue clearance of EVs is typically complete within 6 to 48 h, depending on factors such as EV type, surface modifications, and the methods employed for their isolation and purification [52,53].

Despite their similarities, recent studies have shown that EVs possess superior capabilities for protecting and delivering various therapeutic payloads, compared to lipidic formulations [49,50,54]. As a result, EVs are emerging as a novel and promising natural drug carrier system compared to their competitors. Below, we review the pros and cons of using EVs as drug delivery vehicles and discuss strategies to enhance their drug-loading efficiency and targeting capabilities. For a more detailed comparison of the benefits and drawbacks of using EVs versus liposomes, we suggest recent reviews by van der Koog et al. [55] and van der Meel et al. [49].

### 3.1. Pros and Cons of EVs as Drug Delivery Vehicles

EVs offer several compelling advantages over synthetic drug delivery systems, making them attractive candidates for therapeutic applications. One of the primary benefits of EVs lies in their biocompatibility. As natural components derived from biological systems, EVs are less likely to trigger adverse immune responses, compared to synthetic nanoparticles, thereby improving their profile [56]. Furthermore, EVs exhibit remarkable versatility in cargo encapsulation. They can carry a diverse array of biomolecules, including proteins, lipids, and nucleic acids, and can simultaneously deliver multiple therapeutic agents [57]. In terms of quantity and variety in loading capacity, EVs outperform other systems, such as liposomes, leading to high throughput. This unique feature enhances the therapeutic efficacy of EV-based delivery systems, surpassing traditional carriers like liposomes [49,58].

Additionally, EVs are amenable to surface modifications, which allow for the attachment of specific ligands and targeting ligands. Such modifications can direct EVs to particular tissues or cells, thereby optimizing the therapeutic impact and minimizing off-target effects [59,60,61,62,63,64,65]. The natural lipid bilayer of EVs also serves as a protective barrier, preserving the stability and activity of the cargo during transport and ensuring effective delivery to the target site [56]. Finally, EVs can be produced on a large-scale using cell culture methods, which has the potential to meet clinical demand, especially as research continues to refine scalable and cost-effective production techniques [66]. Currently, large-scale production is performed using cell culture bioreactors, such as EVs from human cardiac progenitor cells [67] or EVs derived from human mesenchymal stromal cells [68].

Despite these advantages, several challenges must be addressed to fully harness the therapeutic potential of EVs. The inherent heterogeneity of EVs, in terms of size, cargo loading, surface markers, and biogenesis pathways, poses significant challenges for their clinical and preclinical development. This variability complicates the standardization of production, characterization, and dosing protocols, making it difficult to ensure consistent therapeutic efficacy and regulatory compliance across different applications.

EVs are preferably purified using density-, size-, and shape-based separation techniques, such as ultracentrifugation or size-based filtration through membranes or porous materials, including ultrafiltration and size-exclusion chromatography (SEC). These methods are favored for their balance between yield and quality but can be tedious and not well suited for large productions. Alternative approaches, like precipitation, can also be utilized, offering higher yields at the cost of lower purity and higher inter-batch variabilities. In contrast, affinity-based and microfluidic methods provide superior purity but are often limited by scalability challenges and higher costs [62].

One significant issue is the difficulty in achieving large-scale production while maintaining a consistent quality and yield [66]. Although efforts to develop a good manufacturing practice (GMP) grade method [67] and implement bioreactor culture systems to scale up and add supplements [68] are underway, the work is still ongoing.

Additionally, although EVs can be engineered to enhance their targeting capabilities, their immunotoxicity and bioavailability remains a concern. EVs are often distributed systemically, and, without precise targeting strategies, they might be uptaken by non-target tissues, thereby reducing their effectiveness [69,70]. On the other hand, the use of exogenous peptide, antibody or synthetic molecule fragments as targeting moieties or surface modification can also induce a certain degree of immune response that has to be controlled [63,71].

Once produced, the stability of EVs under various environmental conditions poses a challenge for storage and transport as they may lose functionality over time [72,73]. Another critical hurdle is that the regulatory framework for EV-based therapies is still under development, and relies on existing regulations for biologics and cell therapies, which may delay their approval in the clinical field [2]. Continued research is essential to overcome these obstacles, particularly through innovations in EV engineering, characterization, and isolation techniques, to realize their full clinical potential.

### 3.2. Natural EVs for Therapeutic Uses

EVs used in therapeutic contexts can be classified as natural or engineered. Natural EVs are unmodified and derived from cell types that possess inherent therapeutic potential. For example, EVs derived from mesenchymal stem cells (MSCs) have shown promising benefits in tissue regeneration. This is attributed to their natural ability to hone in on sites of inflammation or tissue injury and exert immunomodulatory effects [74,75]. The therapeutic potential and targeting capabilities of these natural vesicles are directly influenced by the biological characteristics of their source cells, which can vary significantly, even among similar cell types [76]. An illustration of this variability is seen in EVs from human adipose tissue mesenchymal stem cells (ASCs), which exhibit higher neprilysin activity compared to EVs sourced from human bone marrow MSCs (BM-MSCs) [77]. Furthermore, BM-MSC-derived EVs have been observed to reduce cell proliferation and induced apoptosis, whereas ASC EVs tend to increase cell proliferation and do not induce apoptotic effects in U87MG glioblastoma cells [78].

In any case, natural EVs hold immense potential for treating a wide variety of diseases, including inflammatory conditions, cardiovascular diseases, neurodegenerative disorders and cancer. Ongoing clinical trials are already exploring the use of natural EVs, derived from MSCs and other sources, for therapeutic applications. For instance, clinical trials such as NCT04384445 and NCT03608631 investigate MSC-derived EVs for treating severe COVID-19 and dry eye syndrome, respectively, highlighting their emerging role in innovative therapies

### 3.3. Engineered EVs for Therapeutic Uses

**Figure 3:**
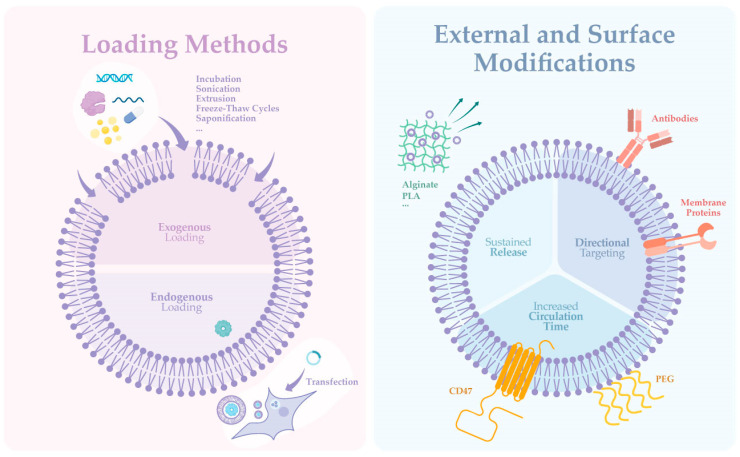
Scheme of the loading strategies and the surface modification possibilities in engineered EVs.

In contraposition to natural EVs, engineered EVs are EVs that have been modified to carry specific bioactive molecules and/or to include targeting moieties that enhance their therapeutic potential. These modifications may also aim to extend their circulation time or enable the sustained release of therapeutic agents (Figure 3) [8,9,55,79].

Regarding loading, engineered EVs can incorporate cargo using endogenous or exogenous methods (Figure 3). In endogenous loading, the cargo is expressed in the producer cell, leveraging the cellular machinery to package the cargo within the EVs. This can be exploited for unmodified therapeutic proteins, as in the case of lysosomal proteins, or modifications can be incorporated to increase the loading efficiency of the cargo. Examples include co-expressing the therapeutic protein with some other protein that is naturally in the EV, like Alix or TSG101 [80], fusion with lysosomal membrane proteins, such as CD9 or CD63 [81,82], or designing proteins fused to plasma membrane proteins, so that they are directed there and associate with lipid rafts [83]. The exogenous loading techniques, such as fusion, osmotic shock, electroporation, sonication, freeze–thaw cycles or saponification, allow for the direct incorporation of the payload [57,74,84,85].

Targeting strategies are critical for improving the therapeutic outcomes of EVs and are studied in both natural and engineered EVs. Exogenously administered EVs can be sequestered by peripheral organs, such as the liver, kidneys, spleen, or lungs. In cases of systemic diseases or when these organs are the target, this might not be an issue; however, in other instances, various targeting strategies can be employed to improve the biodistribution and enhance site-specific accumulations of EVs. Modifications to the EV surface, such as the addition of specific ligands or antibodies, can help direct these vesicles to target tissues or organs more effectively, such as the RVG peptide, that drives EVs to CNS, or antibodies like anti-EGFR, which can target drugs to cancer cells [59,63,64]. Another approach involves choosing the route of administration based on the target organ, which influences the biodistribution of the vesicles; for example, there is a higher EV accumulation in the brain after intrathecal injection, and in the liver and spleen after i.v. and intraperitoneal (i.p.) injections [69]. EVs may be coated with polyethylene glycol (PEG) [60,86,87] or engineered to express immune evasive proteins, like CD47 [61], or reduce the expression of MHC molecules [71] to increase the circulation time and reduce immune clearance.

Moreover, controlled release mechanisms can also be engineered into EVs. These designs allow the vesicle to release their content under specific environmental conditions, such as changes in pH or temperature, providing an active mechanism of release in diseased tissues [62,88,89,90]. Additionally, the integration of polymers, like polylactic acid (PLA) [60,87] or alginate gelatin, helps in creating a sustained-release system, further enhancing the therapeutic efficacy [60,91,92,93].

### 3.4. Loading of Engineered EVs for Protein Delivery

Approximately 18% of preclinical studies have investigated the EV-mediated delivery of peptides or proteins compared to other biomolecules [94]. However, in recent years, protein-based therapeutics have gained increasing interest across many areas of medicine [95]. To date, more than 200 genuine proteins are approved for therapeutic use, with 17 approved in 2023 alone [96]. EVs, as delivery platforms, offer several advantages for these and other protein therapeutics, such as the protection from degradation or immune recognition and the potential for site-specific delivery.

Therapeutic proteins can be loaded into EVs using either endogenous or exogenous methods. In both cases, one of the main challenges is the load of a sufficient quantity of loading into EVs. The choice of EV source and loading method depends largely on the type of protein and the therapeutic context, making a case-by-case approach advisable.

### 3.5. Exogenous Loading of Therapeutic Proteins

Exogenous loading methods generally provide better control over the amount and efficiency of the protein loaded into EVs. This is particularly beneficial for proteins that require high concentrations or do not naturally associate with vesicles. However, exogenous techniques may compromise the integrity of EV membranes, potentially hindering their capacity for effective delivery [97]. Damage to the EV membrane can reduce delivery efficiency, posing a challenge for therapeutic applications [66]. Overall, very different loading efficiencies of proteins have been described for exogenous methods (from 0.4% to 26%), mostly due to differences in the loading technique and the lack of standardization in these processes [98].

Alternatively, exogenous loading can also be achieved by covalently attaching the cargo proteins to the EV, such as conjugating an anti-EGFR peptide to facilitate the accumulation of EVs in EGFR-positive cancer cells, although this approach also has its limitations [99]. Despite these challenges, exogenous loading remains a valuable strategy for modifying EVs to enhance their therapeutic potential, especially in cases where the active principle (i.e., small drugs) cannot be loaded by means of endogenous methods.

### 3.6. Endogenous Loading of Therapeutic Proteins

Compared to exogenous loading, endogenous loading is preferable for complex or large molecules, especially those that are naturally secreted within vesicular systems. Examples include proteins involved in cell signaling or inflammation, or surface proteins [80]. A key advantage of endogenous loading is that it does not require the post-isolation manipulations of EVs, allowing the vesicles to maintain their original biological characteristics. This is particularly beneficial for therapeutic applications, as it ensures that the vesicles retain their functional and cell communication properties. However, the endogenous method often results in variable loading efficiencies, as the quantity of proteins loaded is dependent on the type of protein and on the cell’s capacity to produce and secrete the protein. As a result, very variable loading efficiencies are reported in the literature, with relatively low protein quantities in proteins that are not efficiently secreted into vesicles.

To improve endogenous loading, several engineering strategies have been developed. These include the overexpression of therapeutic proteins fused to surface-associated molecules on EVs, such as Lamp2, CD63, or the N-myristoylation sequence, or to soluble proteins located within the EV lumen, such as Alix or syntenin [80]. Choosing the right anchoring moiety is crucial since loading efficiencies can vary from a few copies of the protein per EV to up to 200 [100]. Since fusing therapeutic proteins to these scaffolds can potentially alter their function, more advanced methods have been designed to release the intact therapeutic proteins. These methods employ triggers like light activation [101,102,103] or small-molecule drugs [104,105] to ensure proper release.

In addition to efficient loading, another important hurdle for protein-loaded EVs is poor endosomal escape. For proteins to reach their intracellular targets, they must successfully escape from endosomes after EV fusion. Vesicular stomatitis virus glycoprotein (VSV-G) is often expressed in the surface of the EVs. This glycoprotein facilitates membrane fusion, upon activation, by endosomal acidification, thereby allowing EV cargo to be released into the cytosol [102,106]. However, its viral origin raises concerns about immunogenicity [107]. As a safer alternative, Syncytin-1 (Syn1), included in the human genome, has been proposed as a non-immunogenic fusogen [108].

The development of these innovative strategies highlights the rapid evolution of endogenous loading approaches, which are paving the way for more efficient, precise, and safer EV-based therapeutics.

### 3.7. EVs for Protein Delivery in LSDs

The current ERTs for LSDs exhibit several limitations, including the short half-life of recombinant enzymes, low bioavailability, sequestration in off-target organs and difficulties in crossing the BBB or penetrating bone. Some patients may also experience the development of anti-drug antibodies (ADAs) and adverse immune responses. Altogether, these issues imply the need for frequent administrations, high dosages and elevated treatment costs [14,47,109]. Given the natural advantages of EVs as protein carriers, different attempts have been made to utilize EVs as a vehicle for lysosomal enzymes (Table 3). The endosomal trafficking pathways of EVs facilitate the delivery of cargo proteins to lysosomes and EVs are naturally enriched in lysosomal proteins. Thus, there are several examples of natural and engineered EVs as therapeutic vehicles in LSDs.

In MPS IVA (Morquio A syndrome) and MPS VII, sEVs from umbilical MSC have been used to deliver GALNS to deficient fibroblasts and to reduce GAG accumulations in MPS VII mouse corneas, respectively [110,111]. In Batten disease, macrophage-derived EVs carrying the enzyme TPP1 have been shown to be effective, both in vitro and in vivo models [69,112]. For FD, alpha-galactosidase A (GLA)-loaded EVs obtained from CHO cells have shown effectiveness in both in vitro and in vivo models without the need for adding any targeting sequence to the endogenously loaded enzyme. Calzoni et al. found that lysosomal β-hexosaminidase, causing Sandhoff syndrome, is naturally associated with EVs. Taking advantage of this natural tropism, these authors overexpressed the α-subunit of the enzyme in HEK293 cells and demonstrated that the HEK293-derived EVs are able to partially correct the enzyme deficiency in Sandhoff patient fibroblasts [113]. Similarly, N-sulphoglucosamine sulphohydrolase (SGSH)-loaded EVs from HEK293 cells have also shown increased enzymatic activity [70]. In GD, sEVs derived from HEK293 have been used to transport the glucocerebrosidase (GBA) enzyme, in this case, fusing with VSV-G, demonstrating that functional enzymes can be appropriately targeted to form endocytic compartments within recipient cells [106]. These studies provide clear evidence of the effectiveness of EVs compared to ERTs.

Interestingly, the use of EVs as carriers of lysosomal proteins has not been limited to soluble hydrolases, opening treatment possibilities beyond ERTs. In cystinosis, sEVs loaded with the transporter protein CTNS have been shown to reduce pathologic cystine accumulation in co-cultured CTNS mutant fibroblasts or proximal tubular cells from cystinosis patients [114,115]. In infantile sialic acid storage disease, sEVs loaded with sialin transporters and mRNA have shown efficacy in sialin deficient fibroblasts [115]. Also, for Niemann–Pick C1, Evox Therapeutics is developing proprietary sEVs designed to deliver functional NPC1 transporter proteins to the affected neuronal cells [116].

**Table 3:** Summary of the EVs used for therapy in preclinical models of LSDs.

Disease	Bioactive Molecule	EV Source	Loading and Modifications	Therapeutic Effects	References
Sphingolipidoses					
Fabry disease (FD)	GLA enzyme	HEK293 and CHO DG44	Endogenous loading	EV-GLA is four times more active (×4) than free enzyme reducing kidney Gb3 deposits, and it is active also in brain	[70]
Gaucher disease (GD)	GBA enzyme	HEK293	Endogenous loading Protein fused with VSV-G scaffold and GFP	GBA containing EVs exerted significantly greater GBA activity (×1.45) than unmodified EVs	[106]
Sandhoff disease	α subunit of β-hexosaminidase enzyme (HexA)	HEK293	Endogenous loading	HexA containing EVs induce-2-fold increment of the enzyme product in patient fibroblasts	[113]
Niemann–Pick C1	NPC1 transporter	MSC	Natural EVs Engineered EVs: Endogenous loading Transporter fused to CD63 or syntenin	Treatment of *Npc*^−/−^ mice with NPC1-loaded EVs reduced cholesterol levels 20–30% in the liver and hippocampus, vs. non-treated or empty-EV treated mice	[116,117]
Neuronal Ceroid Lipofuscinoses (NCL)					
CNL2 (Batten disease)	TPP1 enzyme	Bone marrow-derived macrophages (BMM) and IC21 macrophages	Exogenous and endogenous loading	In vivo EV-TPP1 treatments increased lifespan of Batten mice, especially when combining intraperitoneal and intrathecal administrations	[69,112,118]
Mucopolysaccharidoses (MPS)					
MPS IVA (Morquio syndrome)	GALNS	Human umbilical vein MSC	Natural EVs	Patients’ fibroblasts treated with EVs increase ×16 GALNS activity vs. non-treated fibroblasts	[110]
MPS VII	β-glucuronidase-	Human umbilical vein MSC	Natural EVs (not confirmed directly)	30% reduction in GAG content in corneas of mice receiving MSC transplantation when compared to the untreated control. Effect is attributed to EVs but not confirmed directly	[111]
MPS III (Sanfilippo syndrome)	SGSH enzyme	HEK293 and CHO DG44	Endogenous loading	3-fold increase in enzyme activity of SGSH loaded EVs vs. recombinant enzyme	[70]
Others					
Cystinosis	CTNS transporter protein and mRNA	MSC and Baculovirus-infected Spodoptera cells	Natural EVs and CTNS loaded EVs (fused to CD63) from MSC and CTNS loaded microvesicles from Spodoptera cells	Cystine accumulation is reduced around 60%in patient fibroblasts treated with EV-CTNSs vs. empty EVs. Natural EVs, produced in mammal or insect cells have also a clear effect reducing cystine accumulation, compared to control non-treated cells.	[114,115,116]
Infantile Sialic Acid Storage Disease (ISSD)	Sialin transporter protein and mRNA	Baculovirus-Infected Spodoptera Cells	Endogenous loading	Around 75% of sialin depletion in ISSD fibroblasts after 96 h incubation	[115]

## 4. EVs as Therapeutic Vehicles in Diseases with Neurological Symptomatology

Treating disorders characterized by neurological symptoms poses a significant challenge, primarily due to the difficulty of crossing the BBB. This barrier is a critical obstacle, preventing nearly all therapeutic drugs from effectively reaching the brain [45]. Consequently, there is an urgent need for innovative and efficient delivery systems. In this regard, EVs exhibit unique and promising features, compared to traditional drug delivery systems: they are naturally produced by cells, carry biological cargo that can be tailored to enhance the expression of specific proteins, possess inherent anti-inflammatory properties, and most importantly, have the innate ability to cross the BBB [119,120].

### 4.1. Crossing the BBB

The BBB is a highly specialized structure, which has evolved to protect and maintain CNS homeostasis. It prevents the entry of toxins and pathogens while tightly regulating the endogenous trafficking of essential substances, such as nutrients, hormones, and immune cells [121]. Beyond the BBB, the brain is further safeguarded by two additional protective barriers: the blood–cerebrospinal fluid barrier (BCSFB) and the arachnoid barrier. Both barriers work in concert with the BBB to ensure a tightly regulated environment, which is crucial for proper brain function and homeostasis [122]. The BBB’s effectiveness is attributed to its unique structural properties, which differentiate it from other vascular systems. Endothelial cells within the BBB are non-fenestrated and are connected principally by tight junctions and adherens junctions, limiting the transport of substances via both paracellular and transcellular routes [123,124,125]. The complexity and efficiency of the BBB poses a significant challenge for drug development. For small molecules, simply adhering to Lipinski’s Rule of Five is not sufficient [126,127]. Studies have shown that to cross the barrier via passive diffusion, a molecular weight of less than 400 Da and fewer than eight hydrogen bonds are necessary, a criteria that very few drugs meet [128]. For large molecules, such as proteins, passive crossing is impossible, underscoring the need for specialized delivery strategies.

Over the past few decades, significant research has focused on developing more efficient methods to deliver drugs to the CNS. Various technologies have emerged, including carrier-mediated transport for small molecules, the receptor-mediated transport of proteins, adsorptive-mediated transcytosis (AMT) (using cationic proteins and cell-penetrating peptides) for small molecules and proteins, and nanocarrier-based drug delivery designed for small and large molecules, as well as proteins and genes. These systems include liposomes, polymers, hydrogels, lipid nanoparticles, magnetic nanoparticles and EVs [7,127,128].

### 4.2. EVs as Platforms for Brain Delivery

EVs derived from various sources (different species, tissues and cancerous/non-cancerous cells) can cross the BBB and enter the CNS at different rates, through different mechanisms and with varying selectivity [129]. Currently, there is no systematic and comprehensive understanding of the precise mechanisms by which the EVs cross the BBB; however, evidence from multiple studies provide valuable insights [9,130]. These findings suggest that the transfer of EVs is selective and occurs even under healthy conditions, though it is significantly enhanced in inflammatory states.

#### 4.2.1. BBB Crossing Pathways

The two primary mechanisms by which the EVs could cross the BBB are transcellular and paracellular pathways.

The paracellular mechanism, involving movement through intercellular junctions between endothelial cells, contributes minimally to the total BBB crossing and has not been clearly described as a pathway followed by EVs [131].

**Figure 4:**
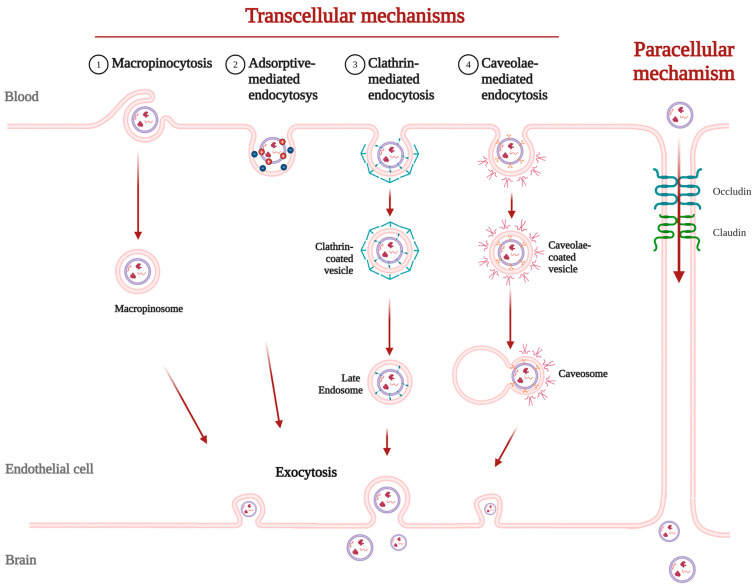
Different mechanisms of crossing the BBB by EVs. Transcellular mechanisms include macropinocytosis, adsorptive-mediated endocytosis, clathrin-mediated endocytosis and caveolin-mediated endocytosis, and imply that EVs cross the endothelial cell to be excreted by exocytosis. Paracellular mechanisms, by contrast, involve EVs moving through tight junctions between endothelial cells.

On the other hand, the transcellular mechanism, that requires transcytosis through the endothelial cells from the apical to the basolateral membrane, is the pathway used by EVs, as well as macromolecules, immune cells, and certain synthetic nanoparticles. This complex process occurs in three sequential steps: endocytosis, intracellular vesicle trafficking and exocytosis [132]. Of these, endocytosis is the most critical step and can occur via four distinct pathways: macropinocytosis, adsorptive-mediated endocytosis, clathrin-mediated endocytosis and caveolin-mediated endocytosis (see Figure 4).

1. 1. Macropinocytosis:

In macropinocytosis, large portions of extracellular fluid are engulfed without the need for specific receptors [133]. This route has been described for EVs isolated from brain-seeking MDA-MB-231 breast cancer cells [134] and sEVs from 293T cells [131]. Although less specific than other BBB crossing pathways, it might contribute to bulk transport, particularly for EVs without specific targeting ligands.

2. Adsorptive-mediated transcytosis (AMT):

Adsorptive-mediated transcytosis (AMT) relies on the electrostatic interactions between the positively charged groups of the drug/delivery system and the negatively charged regions of the membrane on brain endothelial cells [135]. Erythrocyte-derived EVs from PD patients have been demonstrated to surpass the BBB utilizing this mechanism [136].

3. Clathrin-mediated endocytosis (CME):

Receptor-mediated endocytosis includes clathrin-mediated endocytosis (CME) and caveolin-dependent endocytosis. Most EVs seem to follow CME [131,132,134]. The receptors associated with EV uptake and BBB crossing through this pathway include ICAM-1, CD46, or akin [129]. Among them, CD46 has been shown to be specially relevant for facilitating the transport of tumor-derived EVs [137].

4. Caveolae-mediated endocytosis:

In caveolin-dependent endocytosis, caveolae are lipid domains in the plasma membrane that form invaginations upon activation by specific ligand–receptor binding, facilitating intracellular transport [138]. Whether EVs follow this lipid-raft-mediated endocytosis is still controversial. Chen et al. [131] provide supporting evidence, whereas Morad et al. [134] do not. Another mechanism of BBB crossing that commonly occurs via lipid rafts is proteoglycan-mediated transcytosis. Neural stem cell-derived EVs bind heparan sulfate proteoglycans, allowing for their subsequent transcytosis via dynamin activity [139].

Regardless of the transcytosis pathway followed by EVs, inflammation clearly favors this type of transport of EVs across the BBB, as demonstrated by experiments using LPS or TNF-α [9,131,136]. Considering that many, if not all, LSDs are associated with varying degrees of inflammation, this phenomenon likely plays a critical role in modulating the efficiency and specificity of EV-mediated BBB crossing. Thus, the interplay between inflammation and EV transport should be carefully considered when evaluating their potential as drug delivery vehicles in LSDs.

#### 4.2.2. Relevance of the EV Source

Among different EVs explored as vehicles for brain therapeutics, EVs derived from macrophages, MSC, and neutrophils have been investigated for their ability to traverse the BBB and accumulate at sites of inflammation. Specifically, unmodified natural EVs derived from MSC have been extensively investigated in various neurological conditions [140]. In multiple sclerosis (MS) models, MSC-EVs improved motor deficits, decreased brain atrophy, and promoted subventricular zone cell proliferation [141]. In epilepsy, MSC-EVs exhibited neuroprotective and anti-inflammatory properties in vivo [142]. In traumatic brain injury models, MSC-EVs improved spatial learning, reduced neurological deficits, and decreased neuroinflammation across three separate studies [143,144,145]. Apart from their ability to cross the BBB and concentrate in the inflammation sites, MSC-EVs, and the broader MSC secretome, comprises a diverse range of bioactive molecules, such as cytokines, growth factors, and microRNAs [146,147]. These properties are critical in the context of neuroinflammatory diseases, such as multiple sclerosis or traumatic brain injury, and might also be relevant in LSDs [148]. Importantly, since 2019, four different clinical studies have started involving the use of MSC-derived EVs for the treatment of different brain pathologies, confirming the potentiality of these types of carriers [149].

It is well established that the EV cell source significantly influences their BBB-crossing efficiency and CNS targeting. For instance, Haney et al. compared the uptake of EVs originating from three different cell types: primary macrophages (mEVs), neurons (nEVs), and astrocytes (aEVs) [150]. In vitro studies showed the preferential uptake of aEVs by neurons, likely due to the natural communication between neurons and astrocytes. However, in vivo studies using Parkinson’s disease models with acute inflammation revealed higher brain concentrations of mEVs. This phenomenon is probably due to the increased expression of ICAM-1 during inflammation, which facilitates the interaction of macrophage-derived EVs with endothelial cells, their uptake and transport across the BBB [151].

#### 4.2.3. BBB-Targeted EVs

Although EVs possess the natural ability to cross the BBB, enhancing their CNS-targeting potential with specific moieties remains essential. Tailoring the vesicles is a way of taking advantage of receptor-mediated endocytosis, primarily the CME, by utilizing ligands for specific receptors present on the outer membrane of the BBB. A prominent example was the use of the rabies viral glycoprotein (RVG) peptide on the surface of EVs. In 2009, Alvarez-Erviti et al. published a seminal article where mouse dendritic cells were modified to express the RVG fused to Lamp2b, and then EVs were exogenously loaded with the siRNA against BACE-1 by electroporation [63]. The in vivo administration of the engineered EVs drastically reduced BACE1’s mRNA and protein expression in the cortical areas, without eliciting an immunogenic response in wild-type mice. Building on this work, Cui et al. [152], in 2019, tagged natural EVs derived from MSC with the RGV peptide post-isolation through a DOPE-NHS linker. MSC-EVs reduced the levels of pro-inflammatory cytokines, such as TNF-α, IL-β, and IL-6 in Alzheimer’s disease mouse models, supporting the evidence of the intrinsic anti-neuroinflammatory capabilities of the EVs. Other strategies include the use of low-density lipoprotein (LDL) for selective binding to the LDL receptor (LDLR), which is overexpressed on the BBB. Ye et al. incubated EVs with 4F-LDL or 4F-KAL-LDL, where the 4F is an ApoA-I mimetic peptide that facilitates the association of EVs and KLA, a pro-apoptotic peptide with activity in glioblastoma cells [153]. The use of the LDL increased cell internalization, doubled brain accumulation and increased the efficacy of methotrexate-loaded EVs in orthotopic models of glioblastoma. Peptides directly binding the LDL receptors, such angiopep-2, can also be used to increase the ability of EVs to cross the BBB and internalize in glioblastoma cells. Li et al. successfully developed human MSC-EVs expressing angiopep-2 fused to Lamp2 siRNA loaded with glutathione peroxidase 4 (GPX4) and magnetic nanoparticles to trigger ferroptosis in glioblastoma models [154].

#### 4.2.4. The Effect of the Route of Administration

Choosing the right route of administration it is also critical for EV biodistribution and brain accumulation [155]. Systemic administration methods, like oral and i.v. delivery, are limited by the first-pass effect and poor biodistribution. In contrast, the intra-nasal (i.n.) route has been shown to increase the CNS delivery of EVs in a safe and more efficient way, compared to the previously described methods [140,156,157]. Using siRNA-loaded MSC-EVs and the i.n. administration route, Gou et al. elicited significant functional recovery in rats with a complete spinal cord injury [158]. On the other hand, intrathecal (i.t.) injection has already been explored to increase the efficacy of ERT in patients with MPS I, OMS II, and MPS IIIA [19]. This approach bypasses the BBB entirely, allowing for direct drug distribution within the CNS. Notably, in the context of EVs, the i.t. route has been tested in non-human primates using autologous EVs derived from peripheral blood mononuclear cells (PBMCs). This method resulted in an approximately 50 to 100 times higher brain accumulation, compared to the i.p. and i.v. routes, likely due to the reduced peripheral organ accumulation, particularly in the liver, after i.t. injection [159]. Although the potential of EVs loaded with recombinant enzymes to outperform naked recombinant proteins after i.t. administration is promising, the technical challenges of performing this procedure in small experimental animals remain a significant barrier, highlighting the need for continued research and innovative solutions. In the clinical setting, i.t. administration also carries significant risks, including infection, spinal headaches, and nerve damage. Moreover, ERTs via i.t. administration generally show limited therapeutic benefits, and several trials have been discontinued due to poor efficacy or serious adverse events.

### 4.3. EVs for LSDs with Neurological Affectation

Although most of the studies on the therapeutic potential of EVs in LSD were focused on systemic symptoms, some works studied the brain accumulation of EVs in LSD in vivo models or directly aimed at developing CNS-targeted EVs (see Table 4).

#### 4.3.1. Batten Disease or Neuronal Ceroid Lipofuscinoses Type 2 (CNL2)

Batten disease or Neuronal Ceroid Lipofuscinoses Type 2 (CNL2) is a neurodegenerative disease with pediatric onset that results from the deficient activity of the lysosomal enzyme tripeptidyl peptidase 1 (TPP1). The condition is marked by progressive neurodegenerative symptoms, such as delayed language development, seizures, ataxia, movement abnormalities, motor decline, cognitive deterioration, vision loss, and often leads to early death [160].

In recent studies, EVs derived from IC21 peritoneal macrophages carrying enzymatically active TPP1 protein have been demonstrated to target lysosomes in PC12 neuronal cells and CLN2 patient fibroblasts [112]. In this specific study, EVs exogenously loaded by sonication or saponin permeabilization expressed higher enzymatic activity than the EVs obtained from TPP1-transfected cells (endogenously loaded EVs). Moreover, in late infantile neuronal ceroid lipofuscinosis (LINCL) mice, i.p. injections of exogenously loaded EVs remarkably increased mice lifespans, compared to control vehicle-treated animals and reduced astrocytosis, offering more proof of the anti-neuroinflammatory capabilities of the vesicles [112]. Empty EVs induce a subtle increase in mice lifespans, although non-significant. The selection of the cell line for EVs, in these studies, was not spurious. Macrophage-derived EVs are known to have a superior ability to accumulate in the inflamed mouse brain, compared to astrocyte-derived EVs or neuron-derived EVs [150]. The initial study was later consolidated and optimized, addressing different EV administration routes (i.v., i.p., i.t., and i.n.). I.t. administration was the route allowing for a higher EV accumulation in the brain, whereas i.p. injection showed the best biodistribution in peripheral organs. Thus, final efficacy assays with the multiple administration of exogenously TPP1-loaded EVs combined i.t. and i.p. injections to deliver the TPP1 enzyme efficiently in both the CNS and peripheral organs, with relevant increases in CLN2 mice survival [69]. Again, treatment with sham EVs produced subtle, but not significant, increases in the lifespans of Batten disease mice. Finally, the same group recently explored endogenously loaded EVs in LINCL mice [118]. This approach confirmed the therapeutic potential of macrophage-derived, TPP1-loaded EVs. Moreover, the study revealed that TPP1 treatment via EVs not only restored lysosomal function but also induced autophagy. This dual effect contributed to a reduction in neuroinflammation and partial neuronal restoration [118]. Collectively, these findings underscore the potential of TPP1-loaded EVs as a viable therapeutic strategy for Batten disease, offering a multi-faceted approach to mitigate neurodegeneration.

#### 4.3.2. Fabry Disease

Fabry disease (FD) is a progressive LSD caused by a deficiency or complete absence of the alpha-galactosidase (GLA) protein. Although it is not one of the LSDs most severely affecting the CNS, early symptoms at the peripheral nervous systems, such as acroparesthesias, nerve deafness, heat intolerance, hearing loss and tinnitus, are a trademark of the disease [161]. Our group [70] proved that EVs isolated from CHO DG44 dhfr overexpressing the GLA protein exhibited a 10-fold increase in enzymatic activity, compared to soluble GLA from cell supernatants. Remarkably, these EVs showed significantly higher EA than commercially available GLA products. The marked increase in GLA activity within EVs could potentially result from the enhanced stability of these enzymes in a more physiologically compatible environment (acidic pH and membranous scaffold). This environment, characterized by an acidic pH and a membranous scaffold, may better preserve enzyme functionality, similar to the stabilization observed in GLA encapsulated in RGD-functionalized liposomes [162]. Interestingly, no increases in enzymatic activity have been observed for other lysosomal enzymes loaded into EVs via cell transfection when compared to cell lysates, with the exception of the SGSH-enriched EVs presented in the same study.

In vivo experiments using Fabry knockout mice revealed two critical findings. First, high GLA activity was detected in the kidneys. This observation can be attributed to the fact that the distribution of free GLA is primarily regulated by the presence of mannose-6-phosphate receptors (M6PR), which are highly expressed in organs like the liver. In contrast, EV internalization occurs independently of M6PR, suggesting that EV-based therapies could improve enzyme delivery to tissues with lower M6PR expression, such as the kidneys. This property is particularly advantageous in the treatment of Fabry disease, where chronic kidney disease is a prevalent and severe complication. Second, GLA-loaded EVs led to a significant reduction in disease biomarkers Gb3 and LysoGb3 within the brain parenchyma after repeated i.v. administration. Remarkably, this therapeutic effect was achieved even with EVs derived from non-MSC and without any specific surface modifications to facilitate BBB crossing. These findings indicate that even unmodified EVs can reach the brain and exert therapeutic functions, underscoring their potential for treating neurological manifestations of Fabry disease.

#### 4.3.3. Niemann–Pick Type C

Niemann–Pick Type C (NPC) is an atypical LSD due to mutations in genes coding for the NPC1 and NPC2 proteins, which are involved in the cholesterol efflux from the lysosome. The severity of the disorder is defined by the CNS symptomatology, which can vary from the infantile to the late onsets forms [163].

A notable study demonstrated that MSC healthy donor-derived EVs, when i.v. administered to *Npc1*^−/−^ mice, led to significant therapeutic benefits. These included improvements in neurological function, the reversal of neuroinflammation (evidenced by the reduced expression of inflammatory markers such as Ccl3, Ccl5, Cxcl10, and Tnf) and a decrease in both astrogliosis and microgliosis [117]. One plausible mechanism of the EV activity in NPC1 models could be related to the recently discovered role of CD63, a tetraspanin abundantly found in EV membranes, in the cholesterol trafficking [164].

Worth mentioning, Evox Therapeutics, a company based in UK, is actively developing sEVs capable of delivering functional NPC1 proteins to brain cells. The loading of the NPC1 protein into the EVs is eased by the expression of a syntenin/CD63 fusion protein in the membrane [116].

## 5. Challenges and Future Perspectives

A retrospective study by Rahnama et al. identified 46 clinical trials registered up until January 2023 that utilized EVs as treatments, drug delivery systems, or cancer vaccines [165]. Of these, only 10 trials employed EVs specifically for therapeutic purposes, targeting diseases such as COVID-19, cancer, brain infarcts, and tissue regeneration. Expanding on this, Mizenko et al., with broader selection criteria, reported 91 clinical trials investigating EV-based therapies as of December 2023 [166]. Their study highlighted a significant gap in the adoption of engineered EVs, with only 14 studies (15.4%) incorporating these advanced vesicles. This disparity underscores the challenges of translating the promising potential of engineered EVs at the preclinical level into clinical applications. Moreover, current clinical trials have yet to fully explore the engineering capabilities of EVs, instead focusing on strategies such as ultrasound-guided delivery or intrathecal administration to achieve effective therapeutic doses in the central nervous system (CNS) [167].

Transitioning from preclinical studies to the clinical implementation of EV-based therapies, two major challenges arise: large-scale EV production and isolation [168]. As for production, it is well known that cells secrete low numbers of EVs, usually insufficient for a fast and cheap clinical translation: 1 mL of culture medium yields approximately 1 μg of EV proteins, but 10–300 μg protein/mouse are needed in most preclinical studies [169,170]. So far, three approaches have been mainly used to increase the production of EVs in cell cultures: increasing the production of natural EVs by creating and using high capacity “cell factories”, the induction of EV secretion under various cell stressors and strategies based on cell fragmentation with the creation of biomimetic vesicles [171]. Although 2D cultures can also be used for the obtention of clinical-grade EVs [67], most approaches focus on the use of bioreactors, which can produce approximately seven times more EVs per cell, compared to static conditions [172]. The incorporation of microcarriers [68] and hollow fiber systems further enhances production yield, and hollow fibers, in particular, are already being utilized for the production of EVs in clinical trials [173].

Regarding isolation, current methods of purifying EVs are either time-consuming or expensive [174]. Traditional differential ultracentrifugation and density gradient ultracentrifugation are the preferred methods, but they are time and labor intensive with rather low isolation efficiencies [175]. Tangential flow filtration (TFF) provides a more rapid method to isolate EVs but is somewhat limited in its flexibility to isolate specific subsets of EVs [175] and the trend is to combine TFF with other techniques, such immunocapture, affinity, and ion exchange columns and microfluidics [176]. The development of additional methods that enable the improved large-scale, high purity isolation of EVs will surely facilitate the clinical translation of EVs with therapeutic applications in LSDs or other pathologies.

To obtain pharmaceutical products based on EVs, it is necessary to overcome the above-mentioned obstacles and develop a scalable method of producing, isolating and characterizing EVs [177] that comply with Good Manufacturing Practice (GMP). The final objective is to ensure consistency, safety, and quality while complying with all the regulatory standards.

It is also worth mentioning that the regulatory framework for EVs as therapeutic vehicles is still evolving, as they represent a novel and complex class of biologics. Regulatory authorities, such as the U.S. Food and Drug Administration (FDA), the European Medicines Agency (EMA), and other regional bodies, are developing guidelines based on the similarities between EVs and other biologic products, like cell therapies, gene therapies, and nanomedicines. Indeed, EVs may be categorized as biologics, advanced therapy medicinal products (ATMPs), or nanomedicines, depending on their source and therapeutic purpose. The classification also influences the regulatory pathway, as EVs derived from cells (e.g., stem cells, immune cells) may follow different guidelines than synthetic or engineered nanoparticles.

Regulatory bodies emphasize the importance of thoroughly characterizing EVs, including their size distribution, surface markers, cargo (proteins, RNA, lipids), and functional properties. In this regard, the use of standardized methodologies and techniques for EV characterization is crucial. The International Society for Extracellular Vesicles (ISEV) has published several key guidelines and position papers that outline the best practices for the characterization, production, and isolation of extracellular vesicles that can support regulatory compliance [177,178,179]. The implementation of appropriate quality controls is essential, along with a thorough analysis of EV stability during storage.

As a result of this constant and rapid evolution, more than 15 companies are delivering clinical-grade EVs [175] nowadays. Moreover, Evox Therapeutics is already pursuing the use of EVs in LSDs, and more specifically in NPC, thanks, in part, to a partnership with Takeda, one of the big companies providing ERT worldwide. Public–private consortiums are also helping to establish specific guidelines to ensure the rapid clinical translation of therapeutic EVs [180]. Their vision and contribution will surely pave the way for future EV-based treatments in LSD patients.

Looking ahead, advances in the therapeutic EV field will likely emerge from multiple fronts. These include the design of better-targeted EVs engineered to enhance BBB crossing, increased cargo loading capacities (not limited to enzymes but also including transporters), improved production and isolation methods that meet regulatory standards while being more cost-effective, and the standardization of in vivo preclinical assays, including EV dosing and the use of appropriate controls. These developments will be critical to overcoming current challenges and fully realizing the potential of EVs as transformative therapeutics.

## 6. Conclusions

The use of EVs as therapeutic vehicles offers a groundbreaking approach for the treatment of LSDs, particularly those with CNS involvement. Their inherent biocompatibility, ability to traverse the blood–brain barrier, and versatility as drug carriers position EVs as a promising alternative to conventional therapies. Despite the advantages, several challenges must be addressed, including the scalability of EV production, the efficiency of isolation methods, and the establishment of clear regulatory frameworks. Continued research and collaboration between the academia, industry, and regulatory bodies are essential to overcoming these barriers. Achieving clinical translation will require refining the techniques for EV modification and ensuring compliance with GMP standards. In summary, while significant hurdles remain, the therapeutic potential of EVs in LSDs represents a promising frontier that could revolutionize the treatment landscape for these complex disorders.

## Figures and Tables

**Table 2:** Summary of most prevalent lysosomal storage disorders (LSDs) with central nervous system (CNS) involvement. Treatments include enzyme replacement therapy (ERT), hematopoietic stem cell transplantation (HSCT), substrate reduction therapy (SRT), chaperone therapy (CT), PM (proteostasis modifiers), small molecules (SM), gene therapy (GT), BIO (other biological therapies) and others. Adapted from [11,19,46].

Disease	Mutated Gene	Principal Accumulated Substrate	Life Expectancy	Treatments in Use	Treatments in Clinical Phases (Type; Phase; Identification/Drug)
Sphingolipidoses					
Gaucher disease type 2	GBA	Glucosylceramide	<9 months	ERT, SRT, HSCT, SM (Ambroxol)	GT: Phase I/II: NCT04411654; NCT05487599/LY3884961 GT: Phase I: NCT06272149/VGN-R08b
Niemann–Pick type A	SMPD1	Sphingomyelin	<2 years	HSCT	-
Tay–Sachs	HEXA	GM2 gangliosides, glycosphingolipids and oligosaccharides	<4 years	None	GT: Phase I/II: NCT04798235/TSHA-101 GT: Phase I: NCT04669535/AXO-AAV-GM2 SRT: Phase III: NCT04221451/venglustat GZ402671 SRT: Phase III: NCT03822013/miglustat SM: Phase II: NCT05758922/AZ-3102 BIO: Phase I: NCT02254863/DUOC-01
Sandhoff	HEXB	GM2 gangliosides, glycosphingolipids and oligosaccharides	<1 year	None	GT: Phase I/II: NCT04798235/TSHA-101 GT: Phase I: NCT04669535/AXO-AAV-GM2 SRT: Phase III: NCT04221451/venglustat GZ402671 SRT: Phase III: NCT03822013/miglustat SM: Phase II: NCT05758922/AZ-3102 BIO: Phase I: NCT02254863/DUOC-01
Niemann–Pick type C1	NPC1	Cholesterol and sphingolipids	<40 years	SRT (Miglustat), PM (Arimoclomol), SM (N-acetyl-L-leucine)	SM: Phase III: NCT04860960/Hydroxypropyl-beta-cyclodextrin SM: Phase II: NCT05758922/AZ-3102 PM: Phase II/III: NCT02612129/arimoclomol
Krabbe disease (KD)	GALC	Galactosylceramide and psychosine	<1 year (infantile)	HSCT	GT: Phase I/II: NCT05739643; NCT04693598/FBX-101 BIO: Phase I: NCT02254863/DUOC-01 HSCT: Phase II: NCT02171104/allogenic HSCT
			<40 years (late forms)		
GM1 gangliosidosis	GLB1	GM1 gangliosides, KS, glycolipids and oligosaccharides	<1 year	None	GT: Phase I/II: NCT04713475/PBGM01 GT: Phase I/II: NCT03952637/AAV9-GLB1
Metachromatic leukodystrophy (MLD)	ARSA	Sulfatides	<10 years (late infantile)	GT	ERT: Phase II: NCT03771898/SHP611 ERT: Phase I/II: NCT01887938/HGT-1110 GT: Phase I/II: NCT01801709/AAVrh.10cuARSA BIO: Phase I/II: NCT02559830/transduced CD34+ HSC GT: Phase III: NCT04283227/OTL-200 BIO: Phase I: NCT02254863/DUOC-01
			<35 years (juvenile)		
			Decades (adult onset)		
Neuronal Ceroid Lipofuscinoses (NCL)					
NCL	PPT1, TPP1 (CLN2), CLN3, CLN5, among others	Lipofuscin, subunit c of ATPase	<5 years (infantile type)	ERT (only for CLN2)	GT: Phase I/II: NCT05228145/NGN-101 GT: Phase I/II: NCT05791864/RGX-381 GT: Phase I/II: NCT03770572/AT-GTX-502 CT: Phase I/II: NCT05174039/Miglustat SM: Phase III: NCT04637282/PLX-200 BIO: Phase I: NCT02254863/DUOC-01 GT: Phase I: NCT04737460/AAV9/CLN7
			10–15 years (late infantile type)		
			<40 years (juvenile type)		
Mucopolysaccharidoses (MPS)					
MPS I	IDUA	Dermatan sulfate and heparan sulfate	<10 years (Hurler syndrome)	ERT HSCT	BIO: Phase I: NCT05682144/Autologous Plasmablasts (B cells) GT: Phase III: NCT06149403/OTL-203 ERT: Phase I/II: NCT04453085/JR-171 GT: Phase I: NCT06519552/JWK008 GT: Phase I/II: NCT03580083/RGX-111 BIO: Phase I/II: NCT03153319/adalimumab BIO: Phase I/II: NCT03488394/frozen autologous CD34+ hematopoietic stem and progenitor cells genetically modified with the lentiviral vector IDUA LV, encoding for the α-L-iduronidase cDNA, in their final formulation medium
			<40 years (Hurler–Scheie syndrome)		
			Close to normal (Scheie syndrome)		
MPS II	IDS	Glycosaminoglycans	<30 years (neuropathic form)	ERT Modified ERT pabinafusp alfa)	GT: Phase I/II: NCT04571970/RGX-121 GT: Phase II/III: NCT03566043/RGX-121 ERT: Phase I/II: NCT04251026/tividenofusp alfa ERT: Phase II/III: NCT05371613/tividenofusp alfa BIO: Phase I: NCT05422482/GC1123 BIO: Phase I/II: NCT03153319/adalimumab GT: Phase I/II: NCT05665166/Autologous CD34+ HSCs transduced ex vivo with CD11B LV encoding human IDS tagged with ApoEII BIO: Phase III: NCT04573023/JR-141 ERT: Phase II/III: NCT05208281/GNR-055
			50+ years (non-neuropathic form)		
MPS III	SGSH (type IIIA) NAGLU (IIIB) HGSNAT (IIIC) GNS (IIID)	Heparan sulfate	<50 years	None	GT: Phase II/III: NCT02716246/ABO-102 ERT: Phase I/II: NCT06181136/DNL126 GT: Phase I/II: NCT04201405/Autologous CD34+ cells transduced with a lentiviral vector containing the human SGSH gene ERT: Phase I/II: NCT06095388/JR-441 ERT: Phase I: NCT06567769/GC1130A SM: Phase II/III: NCT06614894/ambroxol Hydrochloride
Glycogen storage diseases					
Pompe disease	GAA	Glycogen	<2 years (early onset)	ERT	ERT + CT: Phase III: NCT04138277/AT2221 + ATB200 GT: Phase I/II: NCT03533673/ACTUS-101 GT: Phase I/II: NCT06391736/GC301 GT: Phase I/II: NCT04174105/zocaglusagene nuzaparvovec GT: Phase I/II: NCT04093349/SPK-3006 GT: Phase I: NCT06178432/CRG003 GT: Phase I: NCT06109948/ABX1100
			60+ (late onset)		

**Table 4:** Studies in which EVs have been utilized to target the CNS symptomatology of LSDs.

Disease	EV Source	Therapeutic Agent	Isolation Technique	Main Effects in the CNS	Publication Year
Batten disease (BD)	IC21 macrophages	TPP1 protein	Gradient centrifugation	Increased lifespan in LINCL and CNL2 mice treated intraperitoneally and intrathecally.	2019 [112]; 2020 [69]
	Mouse autologous macrophages	TPP1 protein	Differential ultracentrifugation	TPP1 containing EVs reduced neuroinflammation in CNL2 mice to normal levels. Treatment also increased autophagy.	2023 [118]
Fabry disease (FD)	CHO DG44 dhfr	GLA protein	Polymer co-precipitation	GLA containing EVs reduce c.a. 30% the Gb3 deposits in the brain in GLA KO mice.	2021 [70]
Niemann–Pick Type C (NPC)	MSC from healthy donors	Natural EVs	Polymer co-precipitation	Neuroinflammation indifferent brain areas of *Npc1*^−/−^ mice is reduced to wild type levels after 4 administrations of MSC EVs.	2021 [117]
	Anmion cells, MSC or placental cells	EVs loaded with CD63/syntenin fused NPC1	Filtration and size exclusion liquid chromatography or bead eluate chromatography	EVs reduce c.a. 30% the cholesterol levels in the hippocampus of NPC1 mice	2019 [116]
